# A Study to Investigate the Role of *GULP/ CED 6* Gene in “Eat Me” Signaling in Cellular Efferocytosis and Immunosurveillance

**Published:** 2016-11-30

**Authors:** H Banerjee, B Bazemore, A Barfield, D Crummity, C Krauss, G Payne, J Rousch, V Manglik

**Affiliations:** Department of Natural Pharmacy and Health Sciences, Elizabeth City State University Campus, University of North Carolina, Elizabeth City, USA

**Keywords:** Scavenger cells, Astrocytoma, Fluorescence, Spectrophotometer

## Abstract

In this report, investigations were done to study human *GULP/ CED 6* genes role in presenting cancer cells to scavenger cells. CED 6 SiRNA was used to knock out the gene in Astrocytoma (HTB-12) cell lines to study its effects on expression of various “eat me” signals on these cells including Phosphatidyl serine (PtdSer) expression, nitric oxide (NO) signaling and Leukotrine B4 (LTB4) expression and Caspase 3 activation. Investigations were done by fluorescence microscopy techniques, ELISA assay and colorimetric assays using a standard microplate reader and spectrophotometer. Initial results showed all the above mentioned “eat me” signals were significantly decreased in CED 6 knock out cell lines. Therefore *CED 6* gene must have a role in cancer cell clearance, pathway involved in the cross talk between *CED 6* and other genes in this process is a matter of farther investigation.

## Introduction

The heightened interest in the development of novel anti-cancer drugs that trigger apoptotic death in cancer cells stems from the fact that immediately upon execution of the death signal, the corpse is efficiently removed via specific recruitment of phagocytic cells. Cancer cells need not die in order to be removed by scavenger cells, but could still be effectively phagocytized provided the cell surface expression of specific molecules that strongly engage phagocytic cells is sufficiently enhanced inducing the expression of "eat me" signals on cancer cells which is a novel approach cancer researchers are interested in to "bury alive" these unwanted cells without the untoward effects of chemotherapy-induced apoptosis. Recently Stanford University researchers have shown enhanced cancer cells killing by injecting cancer model mice with CD47 monoclonal antibody and then anti-cancer drugs, since CD47 expresses” do not eat me signals” on cell surface of cancer cells, blocking CD47 exposes the cancer cells and prevent them from hiding the Macrophages and dendritic cells and make them more susceptible to chemotherapy and immunotherapy. Thus success in this field of targeting “Eat me” and “Do not Eat me signaling” in cancer cells has heightened the interest for new investigations in these field [1–3].

The physicochemical properties of cell surfaces change as cells die and are engulfed by neighboring cells (for instance by epithelial cells or fibroblasts), macrophages, or immature dendritic cells. This process is facilitated by expression of several ligands like Leukotriene B4 (LTB4), phosphatidylserine etc., however, cellular stress responses, like release of nitric oxide (NO)which precede cell death are highly diversified, meaning that the history of the pre-apoptotic events that conditions the internal composition and even the surface characteristics of cellular corpses are important for their clearing and phagocytosis. Moreover, the apoptotic and necrotic execution phase itself can involve the variable contribution of distinct catabolic hydrolases including caspases and caspase-independent death effectors, implying that similar morphologies may have been acquired through distinct biochemical routes, thereby influencing the exposure and release of cell death-associated molecules. Following recognition of apoptotic cells, their internalization occurs by the cytoskeletal rearrangement of the engulfing cell. Intriguingly, the signal pathways leading to engulfment are remarkably conserved, with striking similarities between mammals and *Caenorhabditis elegans*. In much simplified terms, two partially redundant genetic pathways involve (with the mammalian homolog in parentheses):

CED-1 (CD91 or LRP1), CED-6 (GULP) and CED-7 (ATP-binding cassette transporter [ABC]) CED-2 (CT 10 regulator of kinase II, CrkII), CED-5 (DOCK180) and CED-12 (engulfment and cell motility (ELMO) [4,5].

CED 6 (GULP) is a signaling adapter protein that functions within engulfing cells downstream of CED 1. The protein contains an N-terminal phosphotyrosine-binding domain (PTB), a central leucinezipper and a proline-rich C-terminal region. The Drosophila, human and mouse homologues have been cloned and all show the same structural organization and a high degree of sequence similarity, suggesting an evolutionary conserved role. Over expression of human CED 6 rescues the engulfment defect of CED-6 mutants in *C. elegans*. In addition, over expression of human CED 6 in mouse J774 macrophages appears to increase phagocytosis of apoptotic cells [6]. These data suggest that at least some of the CED 6 functions may be conserved in mammals.

The specific recognition of the dying cell among the neighboring live cells depends on eat-me signals exposed by the apoptotic cells [7]. To date, multiple eat-me signals have been identified [8]. These include exposure of phosphatidylserine (PtdSer), changes in charge and glycosylation patterns on the cell surface, alteration of ICAM-1 epitopes on the cell surface, and exposure of calreticulin. Among these, the exposure of PtdSer on the outer leaflet of the plasma membrane is the most universally seen alteration on the surface of apoptotic cells [8]. In fact, PtdSer exposure is the best studied and the most accepted definition for calling a cell apoptotic [9].

Leukotriene B4 expression (LTB4), nitric oxide (NO) release are also hall marks of dying cells and helps phagocytes in finding these apoptotic cells for engulfment and clearance [10].

In this study, investigation was done to study the role of GULP in presenting cancer cells to scavenger cells. GFP tagged CED 6/GULP SiRNA was used to knock out the gene in Astrocytoma (HTB-12) cell lines to study and confirmed by immunoblotting and its effects on expression of “eat me” signals by Annexin-V FITC assay for the specific recognition of PtdSer, LTB4 expression by ELISA and cellular stress indicator nitric oxide (NO) signaling by GREISS Nitrite assay.

Our interesting findings are described in this manuscript.

## Materials and Methods

Cell culture: HTB 12 Human Astrocytoma cells were obtained from ATCC (VA, USA) and cultured in L-15 medium in a standard cell culture incubator at 37°C.

Apoptosis was induced by treating the HTB12 cells with TNFalpha for 24 hours and measuring cell death by Trypan Blue assay.

Mammalian GULP GFP tagged SiRNA was obtained from Santa Cruz Biotechnology (USA) and HTB-12 cell lines were transfected by using LIPOFECTIN transfection reagent from Thermo Fischer Corporation (USA). GFP expression was studied by using a Zeiss fluorescence microscope.

GULP knock out HTB12 cell lines were further confirmed by doing a GULP-ELISA assay (Biosource Corporation, USA) using cell lysates.

Nitrite release was determined by mixing 50 µl of culture medium with 50 µl of Griess reagent (1 part 0.1% naphthyl ethylene diamine dihydrochloride in H_2_O plus 1 part 1.32% sulfanilamide in 60% acetic acid in a 96-well microtiter plate. The absorbance at 540 nm was measured on a plate reader, and nitrite concentrations were calculated from a standard curve using NaNO_2_ concentrations between 0.1 and 10 nmol.

Leukotriene B4 (LTB4) ELISA was done using a kit from ASSAY DESIGNS™, USA and with the help of a standard biotek microplate reader.

Phosphatidyl Serine (PtdSer) detection assay was done using the apoptosis detection kit from Clontech Corporation (USA), APOALERT-ANNEXIN V-FITC assay kit, using a Ziess-Fluroscence microscope.

## Results and Discussion

**Figure 1:**
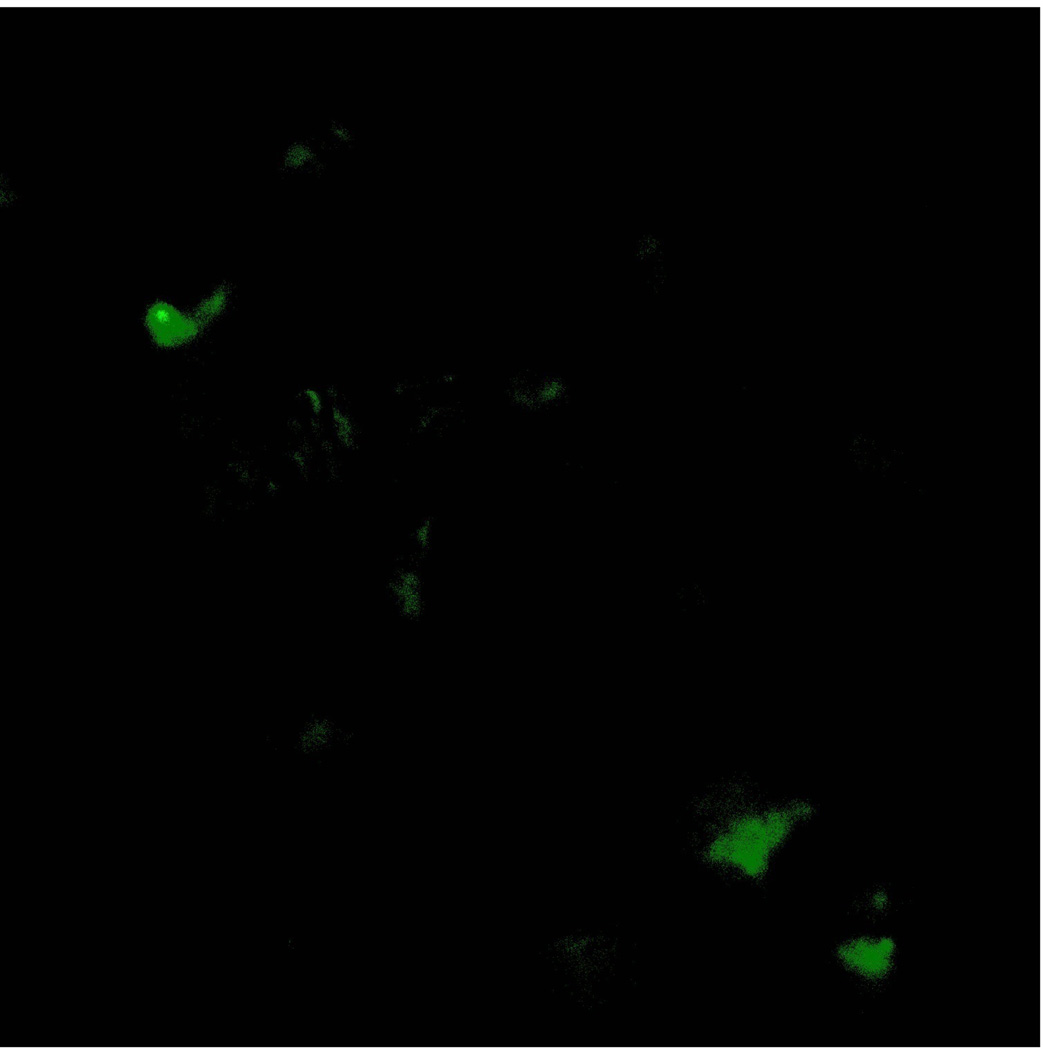
Expression of GFP tagged GULP SiRNA in HTB 12 cells proving effective expression of the construct.

**Figure 2:**
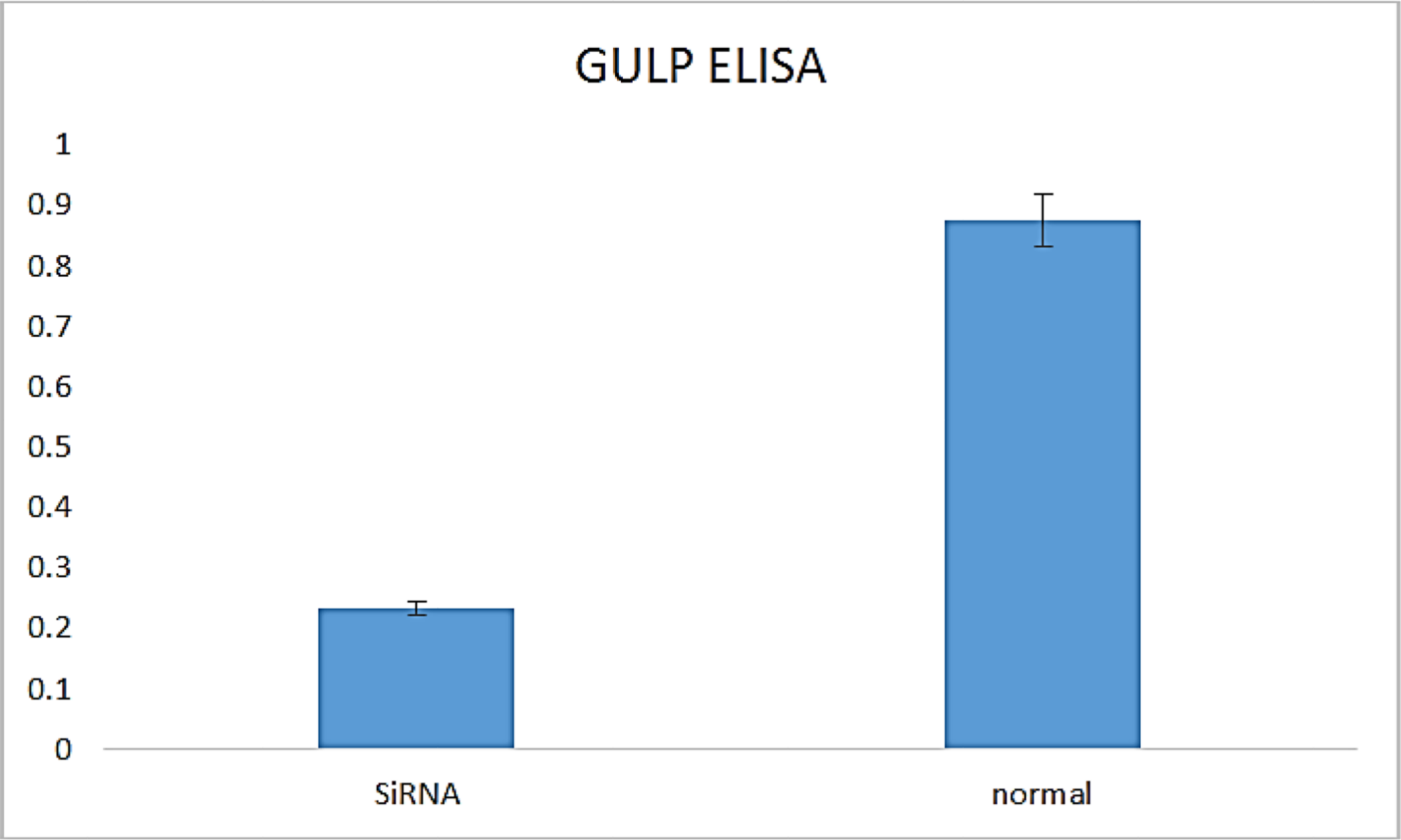
Immunoassay results show significant down regulation of GULP protein by GULP SiRNA knock out.

**Figure 3:**
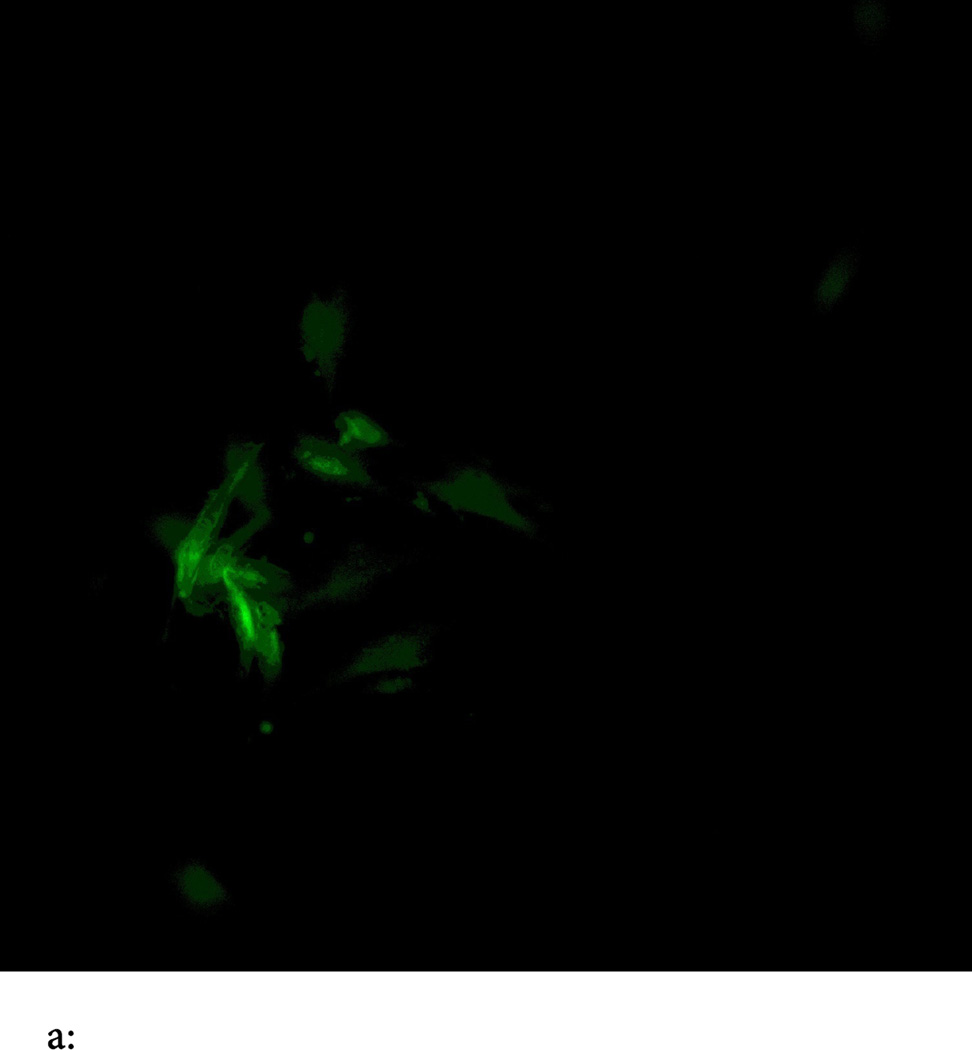

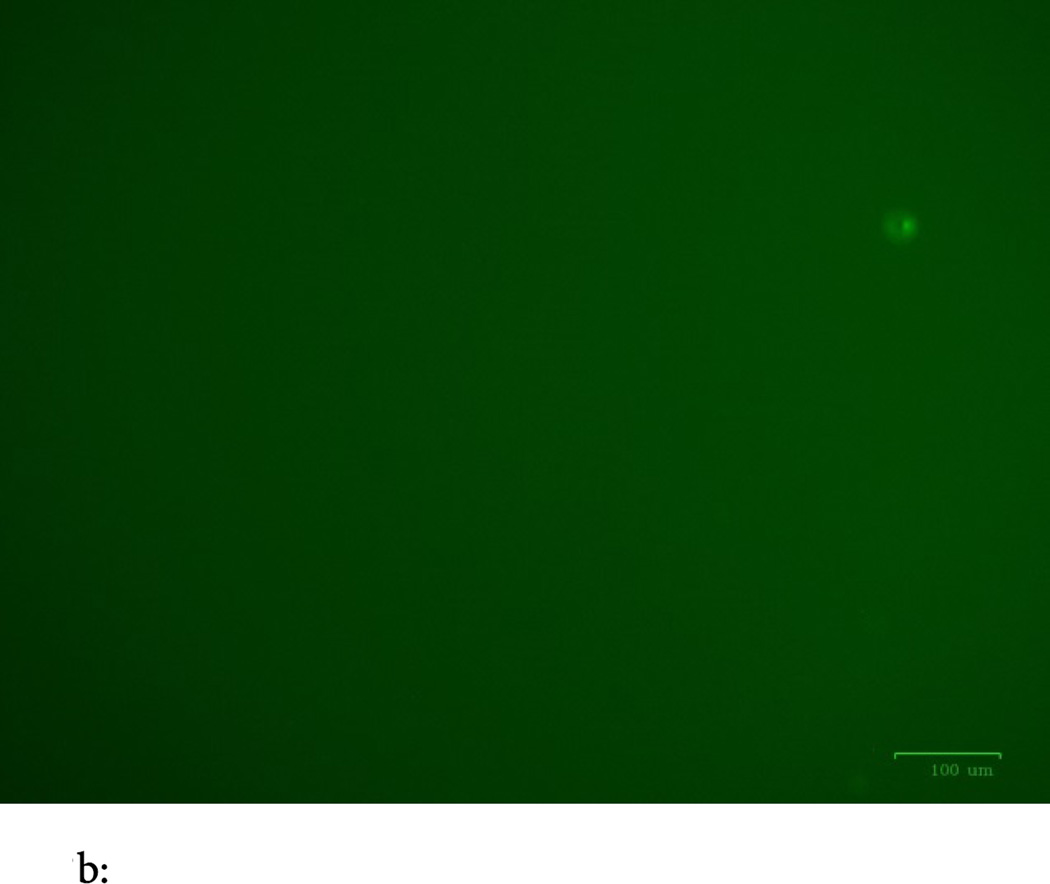
**a:** ANNEXIN V-FITC binding assay to phosphatidyl serine (PS) is showing expression of PS on outer leaflet of cell membrane of 24 hours TNF alpha treated HTB 12 cells, an early hall mark of apoptosis and EAT ME signal. **b:** Annexin V-FITC assay on GULP knock out HTB12 cell lines shows inhibition of PS expression even after 24 hours of TNFalpha treatment.

**Figure 4:**
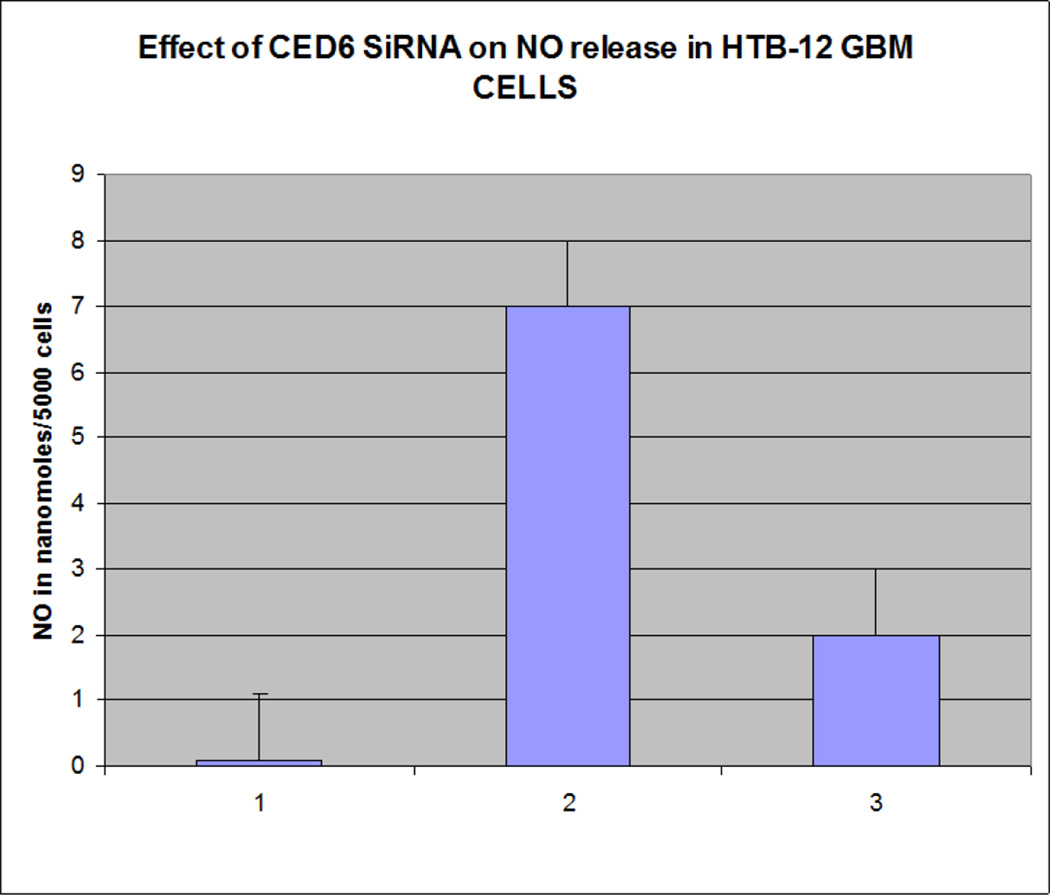
LTB4 ELISA showing LTB4 expression in 1=control HTB12 cells, 2=TNF alpha treated HTB12 cells, 3=GULP SiRNA transfected TNF alpha treated cells. LTB4 expression is significantly down regulated due to GULP inhibition.

**Figure 5:**
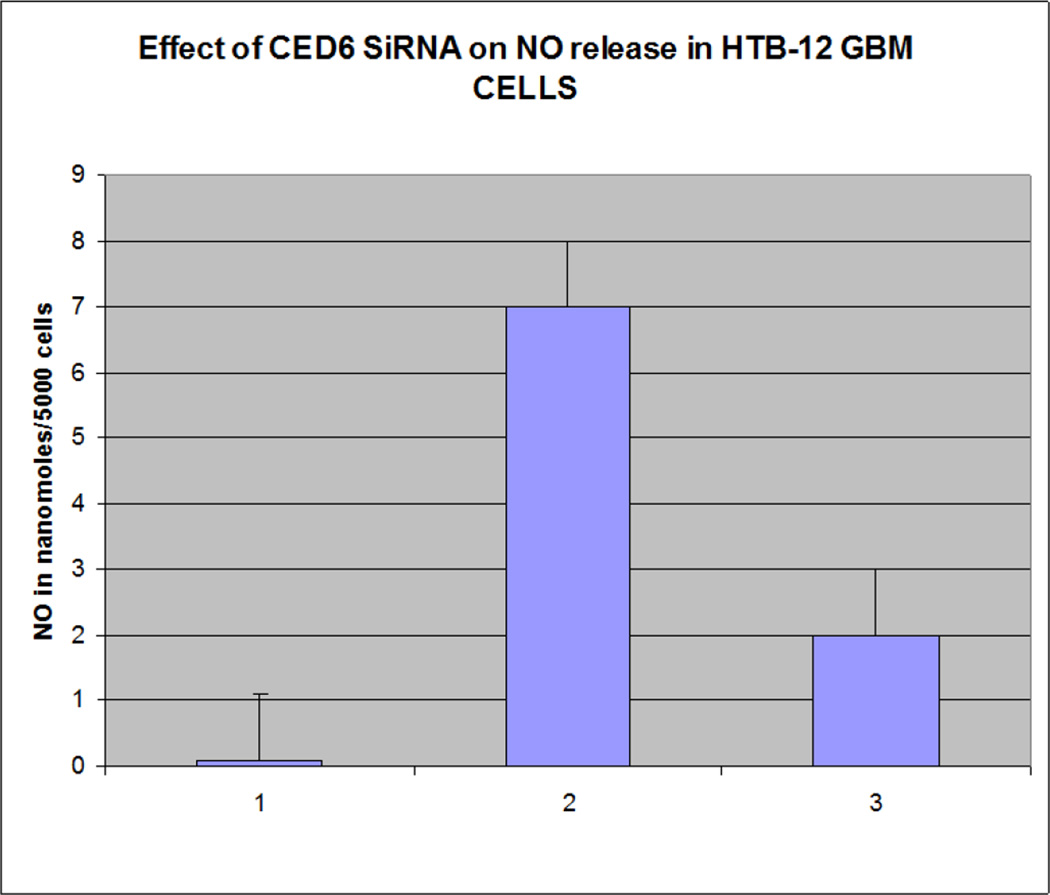
Nitric oxide assay shows GULP knocked out HTB12 cell lines have reduced NO production. Lane 1=control, Lane 2=TNF alpha treated, Lane 3=GULP SiRNA transfected TNF alpha treated HTB 12 cell lines.

Our findings were very interesting and novel, since *GULP/CED 6* gene was found to be involved in upregulation of the EAT ME signals in dying cells and cell corpse clearance, we tested the role of this gene in mammalian cells. We found that knocking out the *GULP* gene (Figures 1 and 2) causes down regulation of several “Eat Me” signals including Phosphatidylserine (Figure 3a and 3b), LTB4 (Figure 4) and stress signal nitric oxide (Figure 5).

Cancer cells hide from macrophages and dendritic cells, however, if these cells could be induced selectively to express the EAT ME signals, then it will be possible to phagocytose them and bury them alive.

Phosphotyrosine binding domain (PTB)-containing engulfment adapter protein (GULP), an adapter protein of LRP1, works on cellular lipid homeostasis by modulating the endocytosis of LRP1 and LRP ligands [11,12]. GULP is a key regulator of LRP1-mediated TGF-β signaling and thereby plays an important role in modulating TGF-β signals in ovarian cells. To support this, microarray studies have shown that GULP is significantly down-regulated in most ovarian adenocarcinomas [13]. Ma et al. [7] presented evidence that GULP is required in ovarian cells to maintain their sensitivity toward TGF-β-induced cell growth arrest, demonstrating the important role that GULP may perform in ovarian cancer progression.

Stabilin-2/FEEL-2/HARE is a large multifunctional glycoprotein that is reportedly a hyaluronan receptor for endocytosis [14,15] and a scavenger receptor that binds bacteria and endocytoses modified low density lipoprotein and glycation end products [12,16]. Stabilin-2 also functions as a membrane receptor involved in the engulfment of apoptotic cells [3], stabilin-2 can stereo specifically recognize PS, and that activation of stabilin-2 by anti-stabilin-2 antibody led to a release of an anti-inflammatory cytokine, transforming growth factor-β, in macrophages, which suggests that stabilin-2 is a bona fide PS receptor for cell corpse clearance in macrophages.

Stabilin-2 contains a large extracellular portion that encodes for seven FAS1 domains, four epidermal growth factor-like domain repeats, a Link domain, a trans membrane region, and a short cytoplasmic region. The cytoplasmic domain of stabilin-2 has NPXY and YXXL motifs (potential binding sites for both PTB and Src homology 2 domains, respectively) that are known to be critical for CED-1-mediated phagocytosis, and *GULP/CED-6* has the PTB domain, which is known to interact with the NPXY motif. The PTB domain of the GULP protein directly binds to the NPXY motif of the cytoplasmic tail of stabilin-2, which provides the evidence for a requisite function of GULP in stabilin-2-mediated phagocytosis.

*GULP* gene, henceforth, could be a novel target for cancer therapy and further investigations needs to be done to study its signaling mechanism and search for compounds or mechanisms that will cause its upregulation in both cancer cells and macrophages for effective clearance of unwanted cancer cells.
